# Job morale of physicians and dentists in Kazakhstan: a qualitative study

**DOI:** 10.1186/s12913-022-08919-x

**Published:** 2022-12-10

**Authors:** Alina Sabitova, Lauren M. Hickling, Medet Toleubayev, Nikolina Jovanović, Stefan Priebe

**Affiliations:** 1 Unit for Social and Community Psychiatry, World Health Organisation Collaborating Centre for Mental Health Service Development, Queen Mary University of London, London, E13 8SP UK; 2 Healthcare Department, Nazarbayev University, Astana, 010000 Kazakhstan; 3 East London NHS Foundation Trust, Newham Centre for Mental Health, London, UK; 4 Department of Plastic Surgery, Astana Medical University, Astana, 010000 Kazakhstan

**Keywords:** Burnout, Healthcare, Job morale, Low-and-middle income countries, Strategies, Thematic analysis

## Abstract

**Background:**

Job morale is thought to be particularly low in Kazakhstan, adversely affecting job motivation, job satisfaction and burnout rates. Previous research suggests that high job morale has a better effect on patient outcomes and care quality. We, therefore, conducted a qualitative study to explore experiences underpinning positive and negative job morale, and to generate potential strategies for improving job morale of physicians and dentists working in public healthcare settings in Kazakhstan prior to the COVID-19 pandemic.

**Methods:**

Three focus groups containing 23 participants and 30 individual interviews were conducted, evidencing respondents' explanations of what affects job morale, and possible strategies to improve it. Data was synthesised using a thematic analysis.

**Results:**

The themes about what influences job morale were: being unfairly rewarded for work; feeling vulnerable and undervalued; poor working styles and practices; and high internal value-based motivation. Various strategies were identified by participants to improve job morale, and these included: ensuring adequate and equitable financial income; improving the current malpractice system; eliminating poor working styles and practices; and creating a shared responsibility for health.

**Conclusions:**

The current study has found that despite prevailing threats, job morale amongst physicians and dentists working in public healthcare settings in Astana have been prevented from becoming negative by their strong sense of calling to medicine and the satisfaction of helping patients recover. Emphasising this rather traditional understanding of the role of physicians and dentists may be a way to improve job morale throughout training and practice.

## Background

‘Job morale’ is a widely used concept both in healthcare and in wider contexts [1]. Although job morale does not have a universally adopted definition, in healthcare research is seen as an umbrella term for various job-related concepts, including job motivation, job satisfaction, burnout and job-related well-being [1]. It was defined that employee needs to be pleased, enthusiastic and comfortable with their job in order to have positive job morale. In contrast, negative job morale was claimed to be present if feelings of displeasure, anxiety and depression prevailed [2]. Current literature suggests that healthcare staff with positive job morale are more likely to provide higher quality care to patients [3], and it has been suggested that improving job morale can improve job performance and/or address inadequate job performance in areas with fewer/inadequate resources [4]. Furthermore, positive job morale is associated with greater retention and higher recruitment of healthcare staff [5]. It has also been suggested that positive job morale is linked with better experiences for patients, contributing to patient-centred care [6, 7]. Negative job morale among healthcare staff, in turn, is linked to poorer patient safety [3], higher levels of self-reported errors [3], poor career engagement [8] and increased healthcare expenditure on staff turnover and sickness absence [9].

Despite that job morale has been reported to differ by professional group [10] and training status [11–13], most studies on job morale in healthcare have targeted either medical residents [14, 15], nurses [16–19] or healthcare staff in general [10, 20–22]. Recent reviews have begun to explore levels of indicators associated with job morale physicians and dentists in Low-and-Middle-Income Countries (LMICs), as well as factors and experiences influencing job morale [23, 24]. This research has showed that job morale in LMICs varied by country and speciality to some extent, but was generally positive, with around 60% of physicians and dentists reporting satisfaction with their job [24]. One of the main factors influencing job morale, is negative experiences such as poor salary or a poor physical or social working environment [23].

Kazakhstan is a country located in Central Asia which became independent in 1991 after the dissolution of the Soviet Union. Since gaining its independence, Kazakhstan has demonstrated strong economic growth, allowing the country to move from the lower-middle-income to upper-middle-income group, according to the World Bank classification [25]. However, economic progress has not been reflected in accompanying improvements in the healthcare system and health-related outcomes. Despite several waves of reforms, reliance on hospital care is still prevailing, and governance remains centralised [26]. Healthcare service delivery is fragmented, and quality of care is impaired by the insufficient staffing levels and limited infrastructure of healthcare facilities [26]. Public healthcare settings in Kazakhstan are funded depending on their level. Financing of outpatient service providers is achieved via a two-level capitation payment system which reflects the number and features of the allocated population, and includes an additional pay-for-performance component [26]. Financing of hospitals is based on diagnoses-related groups, where each diagnosis-related group/ service has a fixed price called a tariff [26]. Per these tariffs, hospitals are reimbursed by the state. Tariff prices for healthcare services are low in Kazakhstan, which results in low salaries for healthcare workers as tariffs are included in the salary costs. In general, physicians’ salaries are formed from a guaranteed basic salary and differential payments allocated depending on state budgetary savings and in accordance with qualification category and exposure to stress [27]. A review by the Organisation for Economic Co-operation and Development (OECD) reported that job morale among healthcare providers in Kazakhstan is low. However, there are limited data supporting this finding or exploring the factors behind the low morale [26].

Considering the previous literature, this study aimed to further explore experiences underpinning positive and negative job morale, and to generate potential strategies for improving job morale of physicians and dentists working in public healthcare settings in Kazakhstan prior to the COVID-19 pandemic.

## Methods

This study report follows the Consolidated Criteria for Reporting Qualitative Studies (COREQ) [28].

### Design

A subtle realist approach was employed in this qualitative study, which stresses that there is ultimately a reality but that human knowledge of this reality is shaped by subjective perceptions [29]. It was appropriate for the context of this study because views about job morale are based on and influenced by participants’ personal experiences and perceptions of these experiences.

It was pragmatically assumed that participants’ personal experiences underpinning job morale and views on what could be done to its improvement will be closely linked and largely overlapped. Therefore, the current study adopted a combination of two methods of data collection—individual, semi-structured, face-to-face interviews and focus group discussions. Specifically, interviews were conducted to collect in-depth personal experiences of job morale, and focus groups to gain a group perspective on what could be done to improve job morale.

### Sampling and recruitment

A multistage stratified purposive sampling method was adopted for the individual interviews. It allowed to ensure the incorporation of a range of experiences via selecting participants groups that "display variation on a particular phenomenon, but each of which is fairly homogeneous” [30]. To begin with, Astana has been chosen as a study site because, as the capital, it allocates all types of state healthcare settings, including polyclinics (primary healthcare settings), regional, city, specialised and national hospitals, which differ in their capacity, infrastructure, financing, and payment schemes. Further, out of physician specialities, psychiatrists have been chosen as representatives of specialised hospitals, surgeons as representatives of regional, city, and national hospitals, and dentists were selected as polyclinics’ representatives. Based on the systematic review and meta-analysis findings that years of experience are likely to be correlated with job morale [24], stratification was advanced by the inclusion of years of practice (0–9 years or 10 + years) as a further stratification category. Thus, physicians could take part if they had qualified with a specialisation in psychiatry, surgery, or dentistry, and were currently employed in public healthcare settings in Astana. Medical students and residents were excluded for this study. Theoretical saturation was achieved by conducting thirty interviews, or in other words “gathering fresh data no longer sparks new theoretical insights” [2]. Although theoretical saturation is conceptually controversial and uncertain [31], it was possible to claim that it had, in fact, been achieved because transcription and familiarisation with data were performed concurrently with the interviews.

To ensure a range of opinions and to identify central themes, maximum variation sampling was adopted for focus group discussions, with the sampling frame designed to increase the variation in healthcare physician specialities, years of practice and employment settings across public healthcare. Thus, physicians and dentists could take part if they were qualified and employed in public healthcare settings in Astana. Furthermore, participants from the individual interviews were invited to attend focus group discussions. It allowed the emerging thematic framework from the interviews’ analysis to be tested with the focus group participants. Three focus groups were planned based on the recommendation that the overwhelming majority of themes would be discoverable within the dataset obtained from three focus groups [32]. The aim was to recruit six to eight participants for each focus group (including physicians who were recruited from the interviews), which is the optimal number of participants to create an adequate group dynamic, giving each participant enough time to discuss their own views [30].

Participants were recruited for interviews and focus groups via adverts disseminated on online social media and WhatsApp. Moreover, participants who had taken part in the individual interviews were invited to participate in the focus group discussions.

### Procedure

At the start of each interview and focus group discussion, participants were provided with the information sheet, introducing study aims and procedures. It was highlighted that participation is voluntarily and anonymous and participants can withdraw from the study at any time. Further, participants were asked to sign an informed consent form, and a short socio-demographic form detailing participants’ specialities, gender, and years of practice was filled out.

AS conducted each offline face-to-face interview in a private office at the participants’ workplaces in the presence of the interviewer and interviewee only between April and July 2019. The interviews were conducted in Russian (*n* = 27), and Kazakh (*n* = 3) and whose duration ranged between 35 and 60 min. Participants received 10,000 KZT (£20) remuneration for their participation. The interview topic guide was developed in English and covered the following areas: 1) general views on job morale in participants’ specialities and professions; 2) specific views on experiences influencing job morale (physical and social work environment, financial and non-financial rewards, work content, and managerial context); 3) general views about the potential impact of job morale on the care provided; and 4) specific views on factors which could potentially worsen or improve job morale. Consistent with the semi-structured interviews, participant leads were pursued by asking follow-up questions to clarify their responses.

All focus groups were conducted by two facilitators (AS and MT) at Astana Medical University during October 2019. The focus groups were conducted in Russian and lasted between 90 and 120 min. AS acted as the main group facilitator and asked the majority of questions. A co-facilitator (MT) assisted the main facilitator by prompting the group during the discussion. Participants received 5000 KZT (£10) remuneration for their participation. The topic guide for the focus groups was also developed in English and consisted of two main parts. Firstly, it included an introduction to the main negative influences on job morale as identified in the individual interviews. Secondly, it provided the framing question and potential probes on what could be done to improve the job morale of physicians and dentists working in Kazakhstan. To ensure the accuracy of the data collected, all interviews and focus groups were audiotaped and field notes were taken.

### Transcription and translation

The transparency and reliability of the study were enhanced by following the recommendations for cross-language qualitative research [33].

AS translated the interview topic guides for individual interviews and focus group discussions into Russian and Kazakh, and MT carried out the backtranslations, ensuring the consistency and validity of the translations.

Interviews and focus groups were transcribed verbatim, and a random subsample were proofread by MT for accuracy. Transcripts of three randomly selected interviews and one focus group were fully were translated into English by the lead author and a translation agency in Astana (agreement 75%). Disagreements were resolved by involving another reviewer (MT), and iterative backtranslation was conducted to ensure reliability and transparency. Further, only quotes used in the study were translated by the candidate and backtranslated by the translation agency in Astana.

### Research governance and ethics

The study received positive opinions by ethics committees at Astana Medical University and Queen Mary, University of London.

### Data analysis

Data analysis was performed in two stages. The analysis of data obtained from individual interviews was followed by the analysis of information gathered from focus group discussions. Thematic analysis was chosen as the analytic strategy in both stages and performed following six recursive phases: familiarisation with data, generating initial codes, searching for themes, reviewing for themes, defining and naming themes and producing the report [34]. A multicultural research team was assembled, spanning a variety of different disciplines. Firstly, AS and MT familiarised themselves with the transcripts and used NVivo (Version 12) to initially code the transcripts. An open inductive or “data-driven” coding approach was utilised, which is highly dependent on and is linked with the data. Furthermore, the inclusivity principle was employed in order to detect as many potential codes as possible [34]. For both individual interviews and focus groups, a random selection of transcripts were selected, and coding frames were created, compared and merged into one by AS and SP. Transcripts were then recoded in accordance with the agreed coding frame by AS. Analysis then moved into more in-depth interpretation of the coded data via collating and merging of codes into themes and sub-themes. During this process, a sufficient level of internal and external homogeneity was reached, ensuring that quotes assigned to the same theme were related, and that quotes assigned to different sub-themes were different, respectively. Themes and subthemes were then arranged and named by AS and SP. Study team members challenged the coherence of developed themes, checked whether they had enough data to support them and ensured the distinctiveness of themes by advising conceptually similar ones be collapsed.

## Results

### Sample characteristics

A total of 30 individual interviews and three focus groups with a total of 23 participants were conducted. Seven interview participants chose to take part in the subsequent focus group, to ensure the emerged frameworks were valid. Sample characteristics are presented in Table 1.

**Table 1:** Characteristics of the sample

Characteristics	Individual interviews (*n* = 30)	Focus groups (*n* = 23)
**Gender (n, %)**		
Female	19 (63.30%)	8 (34.80%)
Male	11 (36.7%)	15 (65.20%)
**Nationality (n, %)**		
Kazakh	26 (86.7%)	20 (87.1%)
Russian	3 (10%)	1 (4.3%)
Belarusian	1 (3.3%)	1 (4.3%)
Ukrainian	0	1 (4.3%)
**Years of practice (n, %)**		
0–9 years	13 (43.3%)	13 (56.5%)
10 years and more	17 (56.7%)	10 (43.5%)
**Speciality (n, %)**		
Dentists	9 (30%)	4 (17.4%)
General Practitioners	0	4 (17.4%)
Specialists	21 (70%)	15 (65.2%)

The results of the current study will be presented in two parts. In the first part, the experiences underpinning positive and negative job morale will be presented. In the second part, the potential strategies for improving job morale will be introduced. Whilst a broad range of themes were noted, there were rarely discrepancies between the accounts offered by participants according to gender, years of practice, and speciality.

### Experiences underpinning positive and negative job morale

Four main themes encapsulated the participant’s experiences underpinning positive and negative job morale (listed in Table 2) these were: 1) being unfairly rewarded for work; 2) feeling vulnerable and undervalued; 3) poor working styles and practices; and 4) high internal value-based motivation.

**Table 2:** Thematic framework for experiences underpinning positive and negative job morale

**1. Being unfairly rewarded for work**
1.1 Workload not reflected by financial rewards
1. 2 Problems accessing professional development
1. 3 Work-life imbalance and intentions to leave
**2. Feeling vulnerable and undervalued**
2.1 Patients’ aggressive behaviour
2.2 Ramifications of the current malpractice system
2.3 Hostile media
**3. Poor working styles and practices**
3.1 Professional, position- and horde-based tribalism
3.2 Disorganised logistical processes
3.3 Insufficient staffing levels
**4. High internal value-based motivation**
4.1 Helping patients recover
4.2 Quantity over quality
4.3 Vocation for being a physician

#### Being unfairly rewarded for work

This theme related to the participant’s experiences of feeling unfairly rewarded for work, which led to a negative effect on job morale. Many participants reported that their remuneration did not reflect their excessive workload, which was felt to be unfair. In addition to this, opportunities to increase their income were inadequate, via payments based on performance indicators and revalidation or increased their workloads further.

“… though you toil down, though you do not toil down, you get a fixed salary. Does it make any sense to work harder if you do not get any more anyway?” (Focus group 3, male, anaesthesiologist)

Opportunities for professional development were also difficult to access. These problems combined often resulted in a professional and personal life imbalance, and increased dentist’s and physician’s intentions to resign.

*“Unfortunately, there is simply no time to learn anything new by reading or attending courses. (…) I spend almost all my time at work because I am employed in three different settings.” (Interview 20, female, psychiatrist)*

It was also recognised that working in healthcare and developing a personal work-life balance was difficult to maintain, with some individuals expressing frustration that their career in medicine has created a barrier to their personal goals outside of work, which was recognised by others.

*“The most frustrating thing about my job is that I live in a polyclinic. I’m an unmarried young lady who is just killing her time here. My parents are worried that I am not interested in starting a family and keep pressuring me.” (Focus group 1, female, GP)*

#### Feeling vulnerable and undervalued

There were numerous reports of participants not feeling valued and at times feeling vulnerable due to patients’ aggressive behaviours, threats to be accused of malpractice at work and the hostile effects of the media.

Even though dealing with the challenging behaviour caused by patients’ physical and mental distress was always part of healthcare providers’ jobs, participants noted a considerable escalation of the aggressive attitudes shown by patients. Some participants reflected on the reasons for aggressive behaviour, and felt that this has been party influences by a financial crisis in Kazakhstan, which had decreased the standards of living.

*“I noticed that our patients and their relatives have become more aggressive than they were before. I think that socio-economic problems cause it. The majority of our patients are from an economically disadvantaged group of the population.” (Interview 19, male, psychiatrist)*

A number of participants also reflected on the shift in the social position of the medical professions in recent years, with some saying that there has been a shift from medical professionals being in a position of ‘social prestige’ to ‘disrespect and intolerance’, further driving aggressive behaviours by patients.

*“The notion that physicians are intellectuals and elite members of society is gone. Our certain social status is gone. That is why patients do not respect us anymore. They do not even speak politely to us.” (Interview 12, male, psychiatrist)*

Questioning the authority and expertise of medical professionals was evident in the form of a dramatic increase in the number of criminal prosecutions against physicians and dentists. The recent introduction of criminal law governing medical malpractice that results in serious, or moderately serious, damage to health or death in Kazakhstan has provided official grounds for malpractice and negligence claims. According to participants, the increased probability of being involved in a malpractice lawsuit has not only weakened the legal certainty of the medical profession but also spread the fear of being accused of such malpractice and has lessened participants’ contribution to patient care.

*“I witnessed how my colleague who was under investigation was handcuffed in front of everyone like some murderer. At that moment, I realised that our profession has become unsafe. I feel like I am afraid now, and I do not want to take responsibility for patients, yet the medical profession demands courage.” (Interview 5, male, surgeon)*

Growing media coverage and challenging medical authority and expertise has also intensified the sense of vulnerability across participants. Participants shared the impression that sensationalist reports, covering sentimental victims’ stories, has been highly detrimental to the status of the medical profession and created negative preconceptions of the healthcare system in general.

*“Now, the medical profession does not have any status or authority. (…) Whatever news you open you can find reports about poor treatment and malpractice. Physicians are guilty in every single clinical incident, even if an investigation is still ongoing.” (Interview 23, female, dentist)*

#### Poor working styles and practices

A common theme among staff was the hierarchical structure of staff within the healthcare setting, and the tensions this can provoke between different levels of staff. These hierarchies seemed to develop organically.

*“Historically, we have hierarchal groups in medicine. For example, auxiliary staff, nurses, physicians, and managers, and so on. Of course, different groups have different powers and authority. I think this is good because there must be a certain despotism in medicine.” (Interview 12, male, psychiatrist)*

Moreover, there are historically determined tribes within the Kazakh ethnic group that belong to three main tribal clusters called ‘*zhuz*’, and which is translated as ‘horde’. The participants admitted there might be a tendency to show favouritism and support towards people from the same *zhuz*, and to demonstrate behaviour according to cultural norms, rather than according to professional standards.

*“The staff in my hospital are from different regions, different tribes, and as you know, those supportive tribal relations are still present in our country. I think it sometimes causes unprofessional behaviour.” (Interview 4, male, surgeon)*

Participants also commented on workplace conditions, noting that resource levels vary depending on employment setting. Despite differences in working conditions, disorganised logistics was a unifying issue for all types of healthcare setting. participants referred to several logistical processes that have been disrupted, including flows of ‘physical’ commodities (pharmaceuticals, essential goods, and equipment), patients and information.

*“There needs to be clear logistical processes. In general, we certainly have them, but details are not taken into account at all, it is such a nightmare. Because of this, every physician and patient suffers.” (Interview 8, male, surgeon)*

Similarly, staffing was generally poor, which predictably leads to a higher workload and more pressure for existing staff.

“In Kazakhstan, the situation is in a deplorable state with psychiatrists. (…) We have only one child psychiatrist for the whole city, who is still hanging on.” (Interview 17, female, psychiatrist)

#### High internal value-based motivation

Despite some frustrations around staffing and resources, many participants reported that their professions helped them to gain a sense of satisfaction and fulfilment, when they helped patients to recover.

“Despite everything I love my job. I have no regrets. I feel like I am making a difference, and it satisfies me. That is the most important thing.” (Interview 20, male, psychiatrist)

However, participants admitted that there had been a shift from quality to quantity of care that had accelerated professional frustrations and endangered their internal motivation to work.

*“In dentistry, we need forty or fifty minutes on average to treat one patient, but we are given only twenty due to increased patient flow. Therefore, we divide the treatment into several appointments. Patients often do not come or postpone appointments, which, doubtless, impacts the quality of treatment.” (Interview 24, female, dentist)*

A number of participants spoke about the perception that medicine is a vocation, and the difficulties this perception can create when healthcare staff request better remuneration for their work.

*“I think that patients sincerely believe that we belong to them, that we should work for nothing, give them all of us and be happy only while helping them. Everyone is so surprised when we are starting to ask for a better payment, better working conditions, or just respect.” (Interview 1, male, surgeon)*

### Strategies for improving job morale

Four main themes were identified, suggesting strategies for improving job morale in medical professionals (see Table 3).

**Table 3:** Thematic framework for potential strategies aimed towards improving job morale

**1. Ensuring adequate and equitable financial income**
1.1 Increasing costs of healthcare services
1.2 Achieving salary transparency
1.3 Improving appraisal and revalidation procedures
**2. Improving the current malpractice system**
2.1 Elaborating criminal law on medical malpractice
2.2 Introducing educational programmes on medical law
2.3 Learning from patient complaints
**3. Eliminating poor working styles and practices**
3.1 Building interprofessional work teams
3.2 Moving towards sustainable supply chain management
3.3 Managing regulatory compliance
**4. Creating a shared responsibility for health**
4.1 Providing revised ethical guidelines for healthcare providers
4.2 Improving patient education
4.3 Revisiting health promotion approaches

#### Ensuring adequate and equitable financial income

A common suggestion for improving job morale, was raising the costs of healthcare services. Healthcare costs in Kazakhstan are particularly low, which means that there is little financial remuneration for healthcare workers. Not only could this have a positive impact on job morale, but on quality of healthcare also.

*“I think that healthcare is very cheap in Kazakhstan. I would say unreasonably cheap. We need to increase tariffs [fixed prices] for our services, so we can get better payment. Currently, our work is not financially valued at all.” (Focus group 1, male, gynaecologist)*

As part of this, it was suggested that salary transparency has become crucial to detecting possible pay discrimination due to the implementation of pay for performance principles. The current lack of accurate information about the remuneration mechanism could obscure existing inequalities and wage gaps.

*“Probably, we need to implement an open-book policy because we have additional payments based on our performance. (…) Currently, I do not understand how my salary is calculated.” (Focus group 2, female, GP)*

Participants suggested that improvements could be made to the revalidation systems, in order to make it clearer and simpler to use. Additionally, it was noted that revalidation assessments should be more relevant to the physician undergoing appraisal.

*“I have recently passed my assessment test to get a qualification category, and there were lots of questions that were not from my speciality. Of course, knowing them is good, but it does not evaluate me as a physician specialising in a certain field. It should be definitely addressed.” (Interview 19, male, psychiatrist)*

#### Improving the current malpractice system

As well as appraisal and revalidation systems being unclear, it was noted by a number of participants that malpractice legislation is similarly unclear. This has contributed to uncertainty about what constitutes ‘improper misconduct’. And because healthcare professionals do not receive training on medical law, many participants felt it would be helpful to elaborate criminal law on medical malpractice.

*“I think the main problem with criminal prosecutions is related to the wording of the law itself. ‘Improper misconduct’ is too vague for medical cases because we might experience unavoidable complications, or we can cause unintentional harm. (…) I think that this law has to be scrutinised, because responsibility for intentional and unintentional harm should not be the same.” (Focus group 3, male, ophthalmologist)*

Participants underlined that educational programmes on medical law should be introduced to prepare professionals equipped to provide specialised advice and representation for both the healthcare providers and patients involved in dispute resolution.

*“Currently, we do not have any professionals who specialise in medical law because it is a completely new field for us. I think relevant degrees or specialisation courses should be offered (…).” (Focus group 1, male, dentist)*

While patient complaints were accepted by service organisations, data from patient complains were rarely used constructively. Participants felt such complaints could be utilised to improve services, rather than penalise staff. The way in which complaints are currently processed does not address the root causes of systemic issues, which limits learning.

“Unfortunately, we do not use patient complaints at all, although it would be possible to analyse them and use this data to improve our services.” (Focus group 2, female, GP)

#### Eliminating poor working styles and practices

As previously mentioned by participants, staff structures within healthcare services were often hierarchical in nature. Participants suggested that instead of keeping the established structures professionals need to collaborate and work with their colleagues rather than following a traditional hierarchy. The purpose of this is to develop interprofessional teamwork. Participants drew attention to the need to extend the roles of nurses while building interprofessional work teams. Physicians and nurses are interdependent and should complement each other’s practices, yet are currently constrained by hierarchical models of working.

*I assume we need to change the way how to work interaction goes within our hospitals. (…) It became possible because the generation has changed. Physicians raised under the Soviet system are retiring, so we really can build a new culture of interactions now.” (Focus group 3, male, surgeon)*

Despite tangible positive changes in supply chain management, there was still certain room for improvement. Participants stressed that the implementation of economic, environmental, and social sustainability principles in supply chain management was needed to ensure development went in the right direction.

*“(…) better logistical processes mean better quality of services, which means better outcomes and better quality of patients’ lives, so we need a system that can work for ages.” (Focus group 3, female, GP)*

The participants in the current study stressed the importance of better regulatory compliance of working hours, employment patterns and better financial compensation to improve job morale.

*“I need to have a second job to provide for my family. But at the same time, I understand that working like that it is unacceptable because I do not see my family. (…) I think some measures should be introduced to control our working schedule. Maybe it will draw attention to our low salaries because we will not be able to ‘artificially’ increase our income.” (Focus group 3, male, anaesthesiologist)*

## Creating a shared responsibility for health

This theme encompassed the need for revised ethical guidelines for healthcare providers in Kazakhstan. It was suggested by participants that these guidelines should clarify ethical principles and how to raise concerns in medical practice.

*“Perhaps our ethical standards are outdated. We are still living by the principles that were developed for the Soviet healthcare system, which was free and for all. (…) I got used to delivering care for everyone and for free, so I am not sure how to say to a patient that he does not have health insurance and he will not get treatment, for example.” (Focus group 1, male, cardiologist)*

Additionally, it was suggested that there should be a shared responsibility for the health of patients, achieved by educating patients about their health and simple first aid and prevention. It was suggested that guidance should be given to patients about how to navigate medical information in the media, in the climate of irresponsible and hostile journalism.

*“I do not believe that we can do anything about our media and their hostile attitude towards us. (…) In this situation, I think we need to explain that not everything being published is true. (…) We can provide something like guidance on how to read health-related articles or something like that.” (Focus group 2, female, ophthalmologist)*

Furthermore, participants noted the urgent need to revisit health promotion approaches as the overall impact of current methods is somewhat limited. It was stressed that health promotion should correspond to the technologically changing environment and demands integrated action by the health sector and the media.

*“I think it is important to understand and promote the fact that we cannot rely purely on the health sector. For example, health promotion programmes should be supported by industries or other stakeholders.” (Focus group 2, female, dentist)*

## Discussion

### Main findings

This study aimed to explore the job morale of health professionals working in public settings in Kazakhstan, and potential strategies for improving it. Participant accounts were largely consistent, regardless of speciality, gender, years of practice and type of state healthcare setting. However, the female participants spoke explicitly about their additional pressures relating to marital status and prioritising of work-life balance.

The findings suggest that where job morale is positive, this is largely due to a high value-based motivation to work in their vocation, and help patients. However, various external pressure threatens the ability of professionals to deliver high-quality care. Participants described being unfairly rewarded for the excessive workload due to meagre income levels and existing problems accessing professional development. Poor financial remuneration often led to participants taking on extra responsibilities, which influenced a poor work-life balance and increased intentions to leave the profession. The perception of medical professions by society and media was also found to lead to poor job morale, as well as poor working cultures and practices. A number of suggestions were made to improve or at least sustain job morale, such as ensuring higher salaries through raised healthcare costs, improving malpractice systems and improving working practices in workplaces.

### Comparison with the literature

Previous research has shown that excessive workloads and a lack of financial reward have been important factors contributing to low job morale of physicians in LMICs [35, 36]. This was predictable, given prior knowledge of the relatively low incomes in the healthcare sector and considering that any increase in income does not correspond to an increase in real income amongst LMICs [37]. Healthcare providers’ remuneration increases with country income group [37], therefore, this issue has only rarely been mentioned in studies of HICs. Multiple employment, while common in healthcare across LMICs, has been suggested to lead to a poor work-life balance, especially in cultures where family (and starting a family) is an important part of a country’s culture [36, 38, 39]. The importance of cultural expectations of family raising, and the pressure of participants to take on multiple jobs and responsibilities was also observed in the present study. Further to this, the findings of the current study reflect the factors found to predict burnout in the context of the COVID-19 pandemic [40]. A recent systematic review aimed to summarise COVID-19 pandemic-specific factors and influences on burn-out and job morale [40]. Similar to the findings of the current study, an increased workload and being unmarried were linked to burn-out [40]. Furthermore, in the context of the COVID-19 pandemic, a wide range of physicians and healthcare professionals were found to be at a higher risk of burnout [41, 42], with a number of different variables predictive of this, such as financial problems, facing violence when caring, being single [41], and facing COVID-19 infection [43], which were also mentioned in the present study.

Increases in income were often dependent on revalidation and appraisals which affected morale. This resembles findings of research among General Practitioners in England, in which General Practitioners claimed that these procedures had a negative impact on job morale and influenced their intentions to leave clinical practice [44]. As in the present study, a sense of feeling undervalued and/or vulnerable to aggressive behaviour was found to contribute to poor job morale in Hispanic nurses working in the USA, which is a High Income Country (HIC) [45]. A further factor linked with poor job morale in this current study was the concern of being sued for malpractice, leading to a sense of vulnerability. A study in Japan found that an increasing number of medical malpractice disputes are being handled through the criminal justice system, although Japan is not widely regarded as a particularly litigious society [46]. This suggests that this factor may be felt by many medical professionals around the world.

Previous research has shown that the interactions and relationships between staff members in healthcare settings were seen as a positive aspect of work in those working in both LMICs [35, 47] and HICs [3, 48, 49], which links to the finding of relationships and interaction positively influencing job morale in the present study. Participants voiced a strong desire to help patients, and whilst altruism is important, it is important to highlight that the medical profession is not purely altruistic, as altruism often creates standards that are un-obtainable [50].

### Strengths and limitations

The current study has the following main strengths. Firstly, to our knowledge, this is the first qualitative study exploring the experiences underpinning positive and negative job morale, and the potential strategies to improve job morale in physicians and dentists working in public healthcare settings in Kazakhstan. Thus, this study allows us to further understand the processes underlying job morale and generate strategies at the local level which should be capable of shaping and driving potential policies and interventions. A second strength relates to its methodological rigorousness and sampling methods, ensuring that the sample included participants from across all types of state healthcare settings, multiple clinical specialities and ranging a vast number of years of clinical experience. A third strength was related to the methodological and participant triangulation, increasing the depth, breadth, and validity of the findings. Methodological triangulation was achieved by adopting two methods of data collection, namely individual semi-structured interviews and focus group discussions. Participant triangulation, in turn, was achieved by inviting participants from the individual interviews to attend focus group discussions. Additionally, the research team included researchers from the culture in which the study was conducted, thereby increasing cultural sensitivity, which is thought to be critical when conducting focus groups with culturally and linguistically diverse samples [51].

A key limitation of the study is the recruitment of participants via social media, which may not be representative of the target population. However, this limitation is somewhat mitigated by the use of a sampling frame, recruiting a range of experiences and specialties. Another limitation was related to translation bias, which is common in cross-language qualitative research. This limitation was mitigated by involving another native speaker reviewer (MT) and a translation agency in Astana, who performed iterative backtranslations to ensure the transparency and reliability of the translation process. Furthermore, the findings may not be generalisable to other cities/regions within Kazakhstan, although training of professionals, societal context and overall health care system are similar, or other LMIC’s with different economic, legislative and healthcare contexts. Finally, in line with a subtle realist epistemology, it should be noted that the present study may be influenced by the first author, who was the interviewer, main facilitator of focus group discussions, and lead analyst.

### Implications

Future studies should systematically assess and understand job morale of health professionals in the Central Asian region in particular and in LMICs in general, beyond Kazakhstan. Such research should also study how health promotion can be utilised and updated to provide patient education and share the responsibility of healthcare with self-management. Due to the cross-sectional nature of this research, future studies may need to examine longitudinal changes in job morale. However, even more important than assessing job morale are initiatives for improving. Our findings, which are largely consistent with other research, have suggested some strategies for achieving this. Such strategies should be implemented, tested and evaluated in different health care settings. The required research may take time and is unlikely to use strictly controlled experimental designs. In order to progress, international co-operation seems essential so that countries can learn from experiences elsewhere, even if contexts may differ.

## Conclusions

The present study found that despite a number of significant threats, job morale of physicians and dentists working in public healthcare settings in Kazakhstan have been prevented from becoming negative by their strong sense of calling to medicine and the satisfaction of helping patients recover. Emphasising this rather traditional understanding of the role of health care professionals may be a way to improve morale throughout training and practice. Ensuring adequate and equitable financial income, improving the current malpractice system, eliminating poor working styles and practices, and creating a shared responsibility for health and care would help physicians and dentists feel fairly treated, heard and appreciated, and would thus sustain or improve job morale. It was noted that job morale is a key indicator of healthcare system inefficiencies, and significantly influences care quality.

## Data Availability

The datasets used and/or analysed during the current study available from the corresponding author on reasonable request.

## Abbreviations

COREQ: Consolidated Criteria for Reporting Qualitative Studies
GP: General Practitioner
HIC: High Income Country(ies)
LMIC: Low and Middle Income Country(ies)
OECD: Organisation for Economic Co-operation and Development
